# ADOLESCENTS’ ATTITUDES ON GRANDPARENT INVOLVEMENT IN CHILD CARE

**DOI:** 10.1093/geroni/igz038.1712

**Published:** 2019-11-08

**Authors:** Katherine E del Rosario, Raphael Ferrer

**Affiliations:** 1 University of the Philippines, Los Banos, Laguna, Manila, Philippines; 2 University of the Philippines Los Banos, Laguna, Philippines

## Abstract

This descriptive associative study focused on the attitudes of selected adolescents toward the involvement of grandparents (maternal grandmother, maternal grandfather, paternal grandmother, paternal grandfather) in childcare. Specifically, it aimed at determining the rate of closeness of these adolescents toward their own grandparents; their attitudes toward the involvement of their grandparents; and the relationship between the students’ closeness with their grandparents and their attitudes toward grandparents’ involvement. A survey was administered to 185 adolescents aged 16 to 22 from the University of the Philippines. Results showed that most of the respondents (43.38%) are either very close or close to their grandparents, especially their maternal grandmother (n = 126). The attitudes of the respondents toward grandparents’ involvement were measured by looking at their rate of agreeableness on 17 statements. Results showed that most of the respondents either agree or neither agree nor disagree (M = 2.60) on grandparents’ involvement. The correlation analysis showed a moderate to high positive linear association between MGM closeness and MGM involvement (r=.72); MGF closeness and MGF involvement (r=.74); PGM closeness and PGM involvement (r=.63); and PGF closeness and PGF involvement. This implies that perceived closeness increases with perceived involvement. The findings of this study emphasized on the role grandparents play in the lives of adolescent grandchildren. Some implications of this study with regards to future research in this field are described.

